# Taurine Supplementation Increases Post-Exercise Lipid Oxidation at Moderate Intensity in Fasted Healthy Males

**DOI:** 10.3390/nu12051540

**Published:** 2020-05-25

**Authors:** Milena Barbon de Carvalho, Camila Fernanda Cunha Brandao, Priscila Giacomo Fassini, Thiago Mantello Bianco, Gabriela Batitucci, Bryan Steve Martinez Galan, Flávia Giolo De Carvalho, Tales Sambrano Vieira, Eduardo Ferriolli, Julio Sergio Marchini, Adelino Sanchez Ramos da Silva, Ellen Cristini de Freitas

**Affiliations:** 1 Department of Food and Nutrition, School of Pharmaceutical Sciences of Araraquara, State University of São Paulo, Araraquara 14801-902, Brazil; milenabcarvalho@hotmail.com (M.B.d.C.); gabibatitucci@gmail.com (G.B.); bryan.mg@hotmail.com (B.S.M.G.); talessv@hotmail.com (T.S.V.); 2 Faculty of Physical Education, State University of Minas Gerais, Divinopolis 35501-170, Brazil; camila.brandao@uemg.br; 3 Ribeirao Preto Medical School, Department of Internal Medicine, University of Sao Paulo, Ribeirao Preto 14049-900, Brazil; priscilafassini@gmail.com (P.G.F.); eferriol@fmrp.usp.br (E.F.); jsmarchi@fmrp.usp.br (J.S.M.); 4 Ribeirao Preto Medical School. Department of Clinical Oncology, Stem Cells, and Cell Therapy. University of Sao Paulo, Ribeirao Preto 14040-907, Brazil; tmbianco88@gmail.com; 5 School of Physical Education and Sports of Ribeirao Preto University of São Paulo, Ribeirao Preto 14040-907, Brazil; flaviagiolo@gmail.com (F.G.D.C.); adelinosanchez@hotmail.com (A.S.R.d.S.)

**Keywords:** Aerobic exercise, fasting, fat oxidation, glycerol, supplementation, taurine

## Abstract

Based on the fact that taurine can increase lipid metabolism, the objective of the present study was to evaluate the effects of different doses of acute taurine supplementation on lipid oxidation levels in healthy young men after a single bout of fasting aerobic exercise. A double-blind, acute, and crossover study design was conducted. Seventeen men (age 24.8 ± 4.07y; BMI: 23.9 ± 2.57 kg/m²) participated in the present study. Different doses of taurine (TAU) (3 g or 6 g) or placebo were supplemented 90 min before a single bout of fasting aerobic exercise (on a treadmill at 60% of VO_2_ max). The subjects performed three trials, and each one was separated by seven days. Blood samples were collected at baseline and after the exercise protocol of each test to analyze plasma levels of glycerol and taurine. Lipid and carbohydrate oxidation were determined immediately after exercise for 15 min by indirect calorimetry. We observed that TAU supplementation (6 g) increased lipid oxidation (38%) and reduced the respiratory coefficient (4%) when compared to the placebo (*p* < 0.05). However, no differences in lipid oxidation were observed between the different doses of taurine (3 g and 6 g). For glycerol concentrations, there were no differences between trials. Six grams of TAU supplementation 90 min before a single bout of aerobic exercise in a fasted state was sufficient to increase the lipid oxidation post-exercise in healthy young men.

## 1. Introduction

Various studies have investigated strategies to reduce body fat [1,2,3,4] to improve the physical performance of exercise practitioners and athletes [5], as well as to enhance the life quality of the population. Taurine is a free intracellular nitrogenous compound derived from methionine and cysteine, mainly found in abundance in seafood [6]. In addition, taurine is present in almost all mammalian tissues [7,8], and is used as a nutritional supplement due to its antioxidant and anti-inflammatory actions [9,10,11] and ability to improve muscle contraction [10]. Finally, taurine can increase the expression of genes related to energy metabolism regulation, specifically, the utilization of lipid substrates, stimulating the lipolysis process [12,13].

Previous studies from our laboratory demonstrated the positive effects of taurine supplementation on lipid metabolism in both rodents and athletes [13,14]. Evaluating the combined effects of taurine supplementation (2%) and physical training, our research group observed a reduction in final body weight, as well as epididymal and visceral fat deposits of obese rats [14]. Recently, De Carvalho et al. [13] verified the effects of acute supplementation of taurine (6 g) before a maximum swimming performance test and confirmed a significant increase of 8% in the plasma glycerol levels. Therefore, this specific taurine dose seems to be a new supplementation strategy for high-intensity efforts, as the increase in glycerol plasma levels may maintain muscle glycogen stores, since lipolysis also releases free fatty acids to be used as an energy source during exercise.

Furthermore, the use of different acute doses of taurine associated with physical exercise has been investigated to improve muscle performance, oxidative stress, and inflammation [15,16,17,18,19]. In contrast, other authors have reported possible modifications in the energetic substrate utilization post-exercise, specifically lipid metabolism [20,21]. These results were not obtained in a fasted state. Exercise practice at low or moderate intensity and a long duration requires a higher proportion of fat oxidation as an energetic substrate when compared to the use of carbohydrate [22]. In this way, the use of taurine supplementation has been shown to stimulate processes involved in the increase in lipolysis and lipid oxidation [23,24].

Since previous investigations have shown the efficacy of exercise per se, as well as the effects of taurine supplementation on lipid metabolism, it would be relevant to question whether the association of both could enhance lipid utilization during low- to moderate-intensity exercise. Moreover, fasting can also modulate lipid mobilization [25,26], especially during exercise that increases adrenaline and decreases insulin blood levels, which promotes higher levels of free fatty acid utilization as a substrate to energy metabolism. Hence, performing aerobic activity in the fasting state could increase fat use when compared to the fed state [27].

Considering the associations among fasting, taurine supplementation, and aerobic activity, our main purpose was to examine whether this combination could promote additional effects on lipid oxidation promoted by exercise itself. However, the best taurine dose to achieve this aim requires investigation. Therefore, the current study evaluated the effects of two different doses of acute taurine supplementation on lipid oxidation levels in healthy young men after a single bout of fasting moderate-intensity exercise.

## 2. Materials and Methods

### 2.1. Subjects and Sample Size

Seventeen active male, healthy subjects (age 24.8 ± 4.07 y; weight 79.02 ± 10.26 kg; height 1.81 ± 0.06 m; BMI 23.9 ± 2.57 kg/m²) were recruited in the School of Physical Education and Sports of Ribeirão Preto–University of São Paulo. The Ethics Committee of the School of Physical Education and Sport of Ribeirão Preto–University of São Paulo approved the study (approval number CAAE 56205516.2.0000.5659), which was performed respecting the Declaration of Helsinki for studies on human volunteers. Written informed consent was obtained from participants before participation. The size of the sample was based on previous studies with exercise and a fasting state that observed significant results, *n* = 15 [28,29]; *n* = 20 [27,30].

### 2.2. Study Design

A double-blind, acute, and crossover study design was conducted, in which subjects participated in three supplementation protocols to determine the magnitude of the plasma taurine response to oral taurine supplementation associated with moderate-intensity exercise in a fasted state. The experimental trials were composed of two doses of taurine (3 g or 6 g) or placebo capsule supplementation, the order of which was determined by a random draw. The participants reported to the lab four times (experimental trials). In the first trial, blood samples were collected, and the participants performed an exhaustive treadmill test to determine the exercise intensity for the subsequent submaximal exercise protocols in the experimental trials (baseline evaluations).

**Figure 1:**
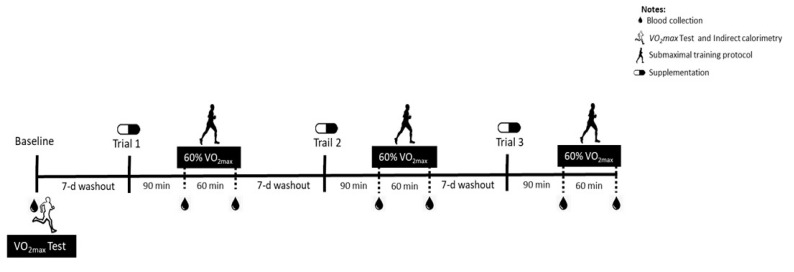
Study design.

In the second, third, and fourth trials, after an overnight fast, the subjects were instructed to ingest the capsules (blind doses) orally. Blood samples were collected 90 min after the supplementation (Pre). Next, the submaximal exercise session was performed for 60 min, and blood samples were again collected post-protocol (Post). A one-week washout was provided between each trial. The supplementation received in each trial was revealed at the end of the complete protocol (after the three experimental trials), when the blind letter was opened. The three trials are described in the Results section as the Baseline, TAU 3 g, Placebo, and TAU 6 g groups (Figure 1).

### 2.3. Taurine and Placebo Supplementation

The supplementation with TAU consisted of 3 and 6 g of taurine [15,31] powder obtained from Ajinomoto CO (Taurine, 99% pure, Ajinomoto CO., INC., São Paulo, SP, Brazil). Starch was used as a placebo (six capsules of 1 g), identical in appearance to the taurine capsules. The taurine and placebo doses were distributed in six capsules to avoid identification of the groups. The 3 g taurine dose was composed of six capsules of 0.5 g, and the 6 g doses of taurine or placebo were composed of six capsules of 1 g. All the capsules were produced by the Industrial Pharmacy of the University Hospital of the Ribeirao Preto Medical School, University of Sao Paulo (HCFMRP/USP).

As previously published [32], maximal taurine blood levels are reached between 60 and 150 min post taurine intake. Therefore, the participants were supplemented 90 min before the exercise protocol to achieve maximal blood levels of taurine during each trial. Participants were requested not to consume taurine food sources, such as energy drinks (Red Bull® or similar), fish, and seafood on the day before the tests.

### 2.4. Maximal Oxygen Uptake (VO_2_ max) Test and Basal Blood Sample Collection

One week before the exercise session, the participants attended the laboratory for baseline sample collection and performed an exhaustive treadmill test (Inbramed Super ATL, Inbramed^®^, Porto Alegre, Brazil) to determine the exercise intensity for the subsequent submaximal exercise bout protocol. After a 5 min warm-up at a self-selected pace with a level treadmill slope, the initial speed was set at 4.5 km/h and kept constant for the first 2 min. Next, the speed was increased by 0.8 km/h every 1 min until 10.5 km/h. The speed then remained constant, and the treadmill slope was raised by 2% every 1 min until exhaustion [33].

The respiratory gas exchange was monitored continuously during the test through an open circuit and automatic, indirect calorimetry (Quark CPET, COSMED®, Roma, Italy), which was calibrated using ambient air and standard calibration gas. The highest O_2_ consumption value obtained during the test was considered as the VO_2_ max. The cardiac frequency was monitored continuously using a heart-rate monitor (Polar M400, Polar®, Kempele, Finland), and subjective perception of effort (PSE) was registered according to the Borg scale [34]. The VO_2_ max of the subjects was classified according to the American College of Sports Medicine [35].

### 2.5. Experimental Trials

In the second, third, and fourth trials, the participants visited the laboratory in an overnight fasted state and were supplemented with taurine or a placebo 90 min before the effort. The exercise protocol was composed of a single bout of running at 60% of their VO_2_ max (controlled by heart rate through the frequency meter) for one hour on a treadmill (the same running speed in all tests) [33]. Heart rate was collected by a heart rate monitor at four moments (in all experiments): pre-exercise, after 30 min of exercise, after 60 min of exercise, and 15 min post-exercise. Blood samples were collected before and after the exercise protocol for further analysis of taurine and glycerol. On the second lab visit, the participants were supplemented with 3 g of taurine, on the third visit with 6 g of a placebo, and on the last visit with 6 g of taurine.

#### 2.5.1. Anthropometric Data

Measurements of body weight and height were performed using an electronic digital scale platform (Filizola^®^, São Paulo, SP, Brazil), with a maximum capacity of 200.0 kg and precision of 0.1 kg. Body mass index (BMI) was calculated according to the formula (kg/m²) = Weight (kg)/[height]². For the assessment of nutritional status, the World Health Organization classification was considered [36]. The body composition was evaluated by skin folds and calculated by Jackson et al. [37].

#### 2.5.2. Biochemical Analysis

Blood samples were collected in 5 mL tubes with heparin (anticoagulant) at baseline, and before and after each experimental trial with all participants respecting an overnight fasting. After the collection, the samples were centrifuged at 1800× *g*, at a temperature of 4 °C for 10 min for plasma extraction. The samples were stored at −80 °C until taurine and glycerol analysis.

#### 2.5.3. Glycerol Analysis

The circulating glycerol assay was performed at rest and immediately after an effort (60 min on a treadmill at 60% of VO_2_ max) by an enzymatic method (glycerol phosphate oxidase) using the Glycerol Assay Kit (Sigma-Aldrich^®^, St. Louis, MO, USA), measured in a spectrophotometer.

#### 2.5.4. Taurine Levels

Plasma taurine was analyzed by High-Performance Liquid Chromatography (Shimadzu, model LC 10AD) by the Deyl, Hyanek, and Horakova method [38], using 99% Taurine (Sigma-Aldrich^®^, St. Louis, MO, USA) as a standard.

#### 2.5.5. Lipid and Glycosidic Oxidation by Indirect Calorimetry

An open indirect calorimeter circuit measured gas exchange (Quark CPET, COSMED®, Roma, Italy), in which the oxygen uptake (VO_2_), carbon dioxide production (VCO_2_), and respiratory exchange rate (RER) were obtained. Before the beginning of the indirect calorimetry, the ambient temperature was measured and recorded by the calorimeter. The monitor was switched on for at least 30 min before the test for proper heating and stabilization. The O_2_ and CO_2_ analyzers were calibrated with standard concentration gas before each determination and periodically validated according to the manufacturer’s specifications. Before flow sensor calibration, a known amount of ambient air was injected into the apparatus through a syringe with approximately 2.5 L of air. After the calibrations, the indirect calorimetry measurement was carried out. Respiratory gases were measured immediately after exercise. Participants wore a face mask covering their nose and mouth, and remained at rest seated in a chair for 10 min. The Respiratory Quotient value (RQ = VO_2_/VCO_2_) provided by the calorimeter in L/minute was used to obtain the oxidation values. The Frayn equation [39] was used to calculate the carbohydrate and lipid oxidation rates. An average of 10 min of measurements (VO_2_ and VCO_2_) was considered to calculate the respiratory quotient (RQ) and substrate oxidation.

### 2.6. Statistical Analysis

After testing the presupposition of normality by the Shapiro–Wilk test, the ANOVA two-way repeated measures or ANOVA one-way repeated measures test was performed, using the GLM module of SPSS (general linear model) for repeated measure analysis and the comparison of unidirectional means for one-way analysis. In cases of group*time interaction, the post hoc Sidak was applied. All analyses were performed with SPSS Statistics™ 20 software (IBM Corporation, Armonk, NY, USA) and GraphPad™ Prism version 5 software (GraphPad Software Inc, La Jolla, CA, USA). Statistical significance was accepted at a level of *p* < 0.05.

## 3. Results

The characteristics of the participants are presented in Table 1. All subjects were physically active, classified as eutrophic according to BMI, adequate according to body fat mass (%), and with a VO_2max_ between average and excellent [35].

Regarding glycerol levels, there was a significant increase after 60 min of the exercise session in all groups supplemented with 3 g, 6 g, or the placebo (comparisons within the group, *p* < 0.001), without group*time interaction (*p* = 0.385). In the comparisons between groups, there was a difference only at the pre-moment (90 min after supplementation), in which the group supplemented with 3 g of taurine presented with a higher level of glycerol compared to the placebo (*p* = 0.008). However, 6 g did not demonstrate differences from the groups supplemented with 3 g and the placebo (*p* = 0.852) (Table 2). Therefore, the changes in glycerol levels are causally related to the exercise session performed, as no differences were observed between the trials post-intervention.

Plasma taurine responses to an acute oral taurine load were observed according to the dose administered. When comparing taurine doses, the TAU 6 g was 94% higher compared to the TAU 3 g post-exercise (*p* < 0.05). Taurine concentrations increased significantly 90 min after supplementation (pre-moment) compared to the post-moment in the groups using 3 and 6 g (within group comparisons, *p* = 0.001 and *p* = 0.017, respectively). In addition, there was a group*time interaction (*p* = 0.003) for the effect of supplementation in the pre- and post-exercise moments, that is, there was a difference between groups in taurine concentration over time, proving that the supplementation was performed in all groups (Table 2). 

Regarding the possible effects of taurine supplementation on heart rate, there was no group*time interaction (*p* = 0.192). There was no difference between groups/supplements; however, there was a difference within groups at different moments. A significant increase in heart rate was observed 30 min after the beginning of exercise in all groups (*p* < 0.001), and this increase was maintained until 60 min after exercise in all groups. At 15 min after the end of the protocol, there was a significant decline in heart rate compared to 60 min (*p* < 0.001) (Table 3). Therefore, a regular physiological response was performed to the submaximal intensity exercise session performed by the healthy young subjects.

**Figure 2:**
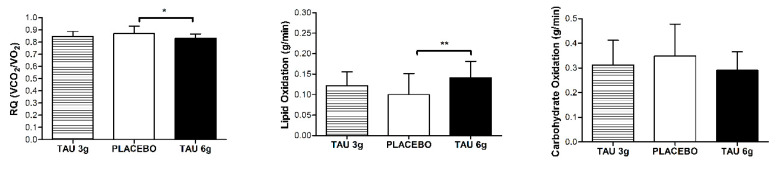
Substrate oxidation after the exercise session. RQ: Respiratory quotient; TAU 3 g: 3 g of taurine supplementation; TAU 6 g: 6 g of taurine supplementation. Symbol represents statistical difference (* *p* < 0.05, ** *p* < 0.01) by ANOVA one-way repeated measures.

To investigate the effects of taurine doses on substrate oxidation, the average of the 10 min of measurements was calculated. We found that TAU 6 g presented an increase of 40% for lipid oxidation when compared to the Placebo group (TAU 6 g = 0.14 ± 0.04 vs. placebo = 0.10 ± 0.05) and a decrease of 4% for the respiratory quotient (TAU 6 g = 0.83 ± 0.03 vs. placebo = 0.86 ± 0.06) (*p* < 0.05). The lipid oxidation of TAU 3 g was 20% higher when compared to the placebo and 13% lower when compared to the TAU 6 g (*p* > 0.05). No differences were observed for carbohydrate oxidation (Figure 2).

**Figure 3:**
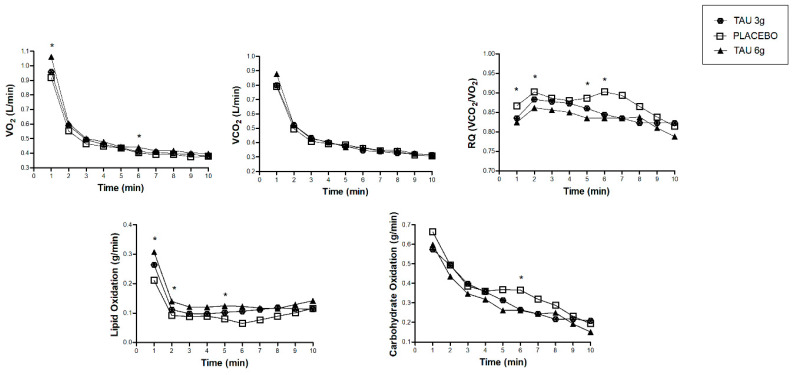
Time-course changes in the oxygen uptake (VO_2_), carbon dioxide production (VCO_2_), respiratory quotient (RQ), lipid oxidation, and carbohydrate oxidation of TAU 3 g, placebo, and TAU 6 g groups. * Difference between TAU 6 g and placebo groups in the time of measures. Statistical difference (*p* < 0.05), by ANOVA two-way repeated measures (general linear model), post hoc Sidak for group*time interaction (respiratory quotient, lipid oxidation, and carbohydrate oxidation).

To further explore the substrate oxidation, Figure 3 shows the time-course measurements of the calorimetry data during the 10 min of evaluation. We showed minute-by-minute differences between the TAU 6 g and placebo groups, such as higher VO_2_ in the first and sixth minutes, lower RQ in the first, fifth, and sixth minutes, and higher lipid oxidation in the first, second, fifth, and sixth minutes for the TAU 6 g group. Carbohydrate oxidation in the fifth minute of measurement was higher in the placebo group compared to the TAU 6 g. Table S1 presents the statistical differences between the times of these measures. There was no difference for the TAU 3 g group, compared to the other groups at the evaluated times. There was a group*time interaction only for the carbohydrate oxidation, lipid oxidation, and respiratory quotient (*p* < 0.001 for all).

## 4. Discussion

The present study addressed the effects of two different doses of acute taurine supplementation on lipid oxidation levels in healthy young men after a single bout of fasting moderate-intensity exercise. The main finding of this investigation was that 6 g of taurine increased lipid oxidation by 38% and reduced the respiratory coefficient by 4.6% when compared to the placebo group. Here, the plasma taurine levels increased at all times after the supplementation period when compared to baseline. These data corroborate the study of Rosa et al. [31] and Zhang et al. [15], which showed a significant increase in the taurine plasma levels after chronic and acute supplementation with 3 and 6 g, respectively. The present study also demonstrated that taurine supplementation did not alter heart rate at rest during and after an exercise session at 60% of VO_2max_ in healthy young men. Our results corroborate another study [19], which demonstrated that supplementation with 1 g of TAU did not change the heart rate during a maximal 3 km time trial performance in trained middle-distance runners.

Taurine has been used to increase lipid oxidation as a predominant energy substrate [13]. This ergogenic effect of taurine may be significant considering the energy potential of lipids, since increased lipid oxidation decreases carbohydrates and preserves muscle glycogen stores, which may improve physical performance and reduce body fat mass levels [40,41,42]. Exercise sessions performed in the fasting state are widely used by endurance athletes to maximize both lipid oxidation and oxidative capacity. Comparing substrate oxidation between two groups of active young men training at 70% of VO_2max_ for 1-1.5h for six weeks after carbohydrate-rich meal ingestion or in the fasted state, Van Proeyen et al. [43] verified a higher contribution of lipids as an energy substrate in the fasted group. Furthermore, Hulston et al. [44] evaluated the lipid oxidation during 90 min at 70% of VO_2max_ (aerobic training) with high or low levels of muscle glycogen in 14 well-trained cyclists, and observed that the fasted group presented with increased lipid oxidation. These results could be explained by the activation of Mammalian 5′AMP-activated protein kinase (AMPK), which is considered an essential energetic sensor of the increase in the AMP/ATP ratio [45]. The activation of this kinase through exercise and/or fasting (i.e., reduced circulating levels of glucose) has been linked to increased glucose uptake, fatty acid oxidation, and mitochondrial biogenesis [46].

Regarding the association between taurine supplementation and exercise, taurine possibly exerts an effect on lipid oxidation through the activation of adenylate cyclase, directly stimulating the production of cyclic adenosine monophosphate (cAMP) or through the increased secretion of catecholamines [47]. According to Watt and Spriet [48], activation of the cAMP cascade, mainly mediated by the catecholamines, is the initial trigger to the increase in lipolysis and lipid oxidation during moderate-intensity exercises. Furthermore, higher taurine levels can modulate the electron transport chain and improve the generation of adenosine triphosphate (ATP) by activating the mitochondrial complex I [49].

Our research group previously investigated the acute effects of taurine supplementation on lipolysis after a bout of exercise. Levels of glycerol, a marker of lipolysis, were evaluated in nine male competitive swimmers after performing a 400 m front crawl effort supplemented with 6 g of taurine or placebo 120 min before performing the effort. It was observed that the glycerol levels were higher in both groups post-effort; however, the TAU supplementation presented with higher glycerol levels (8% of delta variation) than the placebo supplementation [13].

Although the exercise intensity was different from our previous study [13], the results of the present study also suggest a positive effect of taurine on the regulation of lipid metabolism, since higher variation in glycerol levels was observed with the supplementation of 3 g (~4.7%) and 6 g of taurine (~5.7%) compared to the placebo supplementation with a moderate-intensity exercise protocol.

According to Robinson et al. [50], moderate-intensity exercise leads to hormonal and metabolic changes, contributing to increased levels of lipolysis and fat oxidation. When moderate exercise is performed in a fasted condition, there is a decrease in insulin levels (anti-lipolytic hormone) and an increase in glucagon and catecholamines, enhancing free fatty acid levels in the circulation [51]. Therefore, the increased glycerol levels observed in the present study in both groups showed that the moderate-intensity exercise protocol was effective in promoting the availability of lipids as an energy substrate when comparing the post-exercise versus basal state.

However, contrary to our results, Van Loon et al. [52] observed an increase in the blood glycerol concentrations with the same exercise intensity, but with a higher exercise volume (i.e., 120 min) in trained male cyclists. This finding demonstrates the importance of both the intensity and volume of exercise to stimulate the lipolysis process [53].

To the best of our knowledge, this is the first study on the acute effects of different doses of taurine supplementation associated with moderate-intensity exercise in the fasted state. Although no synergistic effect was observed between exercise and the acute doses of taurine supplementation for glycerol levels, fat oxidation increased by 40% with 6 g of taurine (*p* < 0.05), suggesting that a higher dose was necessary to stimulate lipid metabolism. Furthermore, a decrease of 4% was observed in the respiratory quotient when compared to the placebo group, suggesting a relative intensification in lipid and beta-oxidation [54]. Moreover, our results showed a glycerol threshold of around 0.40 µm/dL for both TAU supplementation doses, making it evident that the increase in glycerol levels was an effect promoted by the exercise session; however, this was in association with the results of the lipid oxidation, which were superior to the placebo. Thus, our data indicate that taurine promoted an additional effect on lipid oxidation.

**Figure 4:**
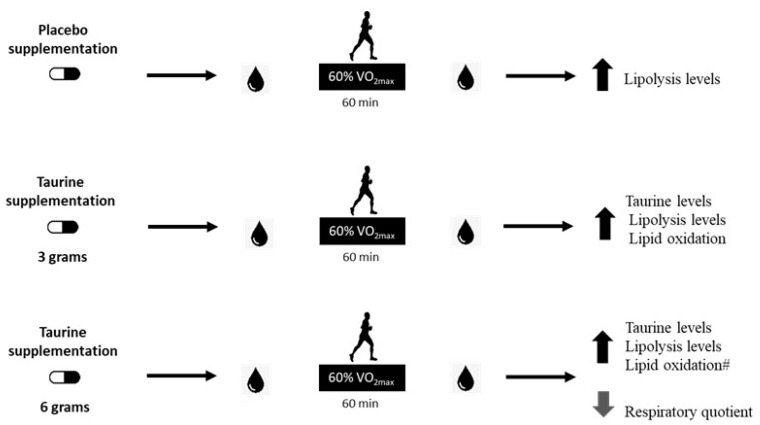
Main findings of the present investigation. Effect of the supplementation of different doses of taurine was evaluated following a bout of exercise in the fasted state, and it was observed that a moderate-intensity exercise session similarly increased lipolysis levels in all trials. Additionally, supplementation with 3 g increased taurine blood levels post-exercise when compared to placebo supplementation. The supplementation with 6 g increased taurine plasma levels and lipid oxidation, and decreased the respiratory quotient post-exercise when compared to placebo supplementation, and lipid oxidation was 27% higher than TAU 3 g (#), showing a greater impact on lipid oxidation than the 3 g in healthy young men.

In summary, this study partially confirmed our hypothesis, since the taurine dose of 6 g improved lipid oxidation when associated with moderate-intensity exercise during the fasting state. Although not significant, the use of 3 g of taurine increased lipid oxidation by approximately 18% in comparison with the placebo trial. Therefore, a dose between 3 and 6 g may show promising results to trigger lipolysis and lipid oxidation processes. Future investigations should evaluate supplementation approaches between 3 and 6 g of TAU to find a better dose response, and thereby, exert an additive effect on the use of fat as the predominant energetic substrate during physical exercise of moderate intensity in a fasting state. Figure 4 summarizes the main findings of the current study. 

## Figures and Tables

**Table 1:** Characteristics of participants.

Characteristics	Mean ± SD
Age (years)	24 ± 4
Weight (kg)	79 ± 10
Height (m)	1.80 ± 0.1
BMI (kg/m²)	23 ± 2
Fat mass (%)	14 ± 4
Fat mass (kg)	11 ± 4
Fat-free mass (kg)	68 ± 7
VO_2max_ (mL/kg/min)	48 ± 8

Data are means ± standard deviations. BMI: body index mass.

**Table 2:** Plasma taurine and glycerol concentration between evaluations.

	Glycerol (µm/dL)			Taurine (µmol/L)		
Groups	Pre	Post	*p*-Value	Pre	Post	*p*-Value
Placebo	0.29 ± 0.01 ^a^	0.37 ± 0.08	<0.001	16 ± 2 ^a^	16 ± 3 ^a^	0.573
TAU 3 g	0.31 ± 0.01 ^b^	0.41 ± 0.05	<0.001	195 ± 57 ^b^	143 ± 37 ^b^	0.001
TAU 6 g	0.30 ± 0.03 ^a,b^	0.40 ± 0.04	<0.001	411 ± 175 ^c^	278 ± 134 ^c^	0.017

Data are means ± standard deviations. Different letters (a, b) represent statistical difference between groups/supplementation at each moment (*p* < 0.05). The *p*-value represents statistical difference within groups (pre and post), by ANOVA two-way repeated measures (general linear model), post hoc Sidak for group*time interaction in plasma taurine concentration. TAU 3 g: 3 g of taurine; TAU 6 g: 6 g of taurine. Pre: 90 min after the supplementation; post: 60 min after the submaximal training protocol.

**Table 3:** Heart rate (bpm/min) measures during exercise sessions.

Groups	Pre	30 min	60 min	15 min-Post	*p*-Value
Placebo	65.2 ± 3.9^c^	114.57 ± 5.2 ^a^	117.5 ± 2.9 ^a^	77.2 ± 7.5 ^b^	*p* < 0.001
TAU 3 g	69.3 ± 2.4 ^c^	115.2 ± 4.0 ^a^	117.4 ± 6.3 ^a^	76.7 ± 5.2 ^b^	*p* < 0.001
TAU 6 g	68.0 ± 8.4 ^c^	117.0 ± 2.1 ^a^	118.8 ± 6.1 ^a^	75.5 ± 5.3 ^b^	*p* < 0.001

Data are means ± standard deviations. Different letters (a, b, c) represent statistical difference within groups, between time-points (*p* < 0.05). *P*-value represents statistical difference within groups by ANOVA two-way repeated measures (general linear model). Pre: immediately before exercise. 30 min: 30 min after starting exercise. 60 min: immediately after exercise. 15 min post: 15 min after the end of the exercise.

## Supplementary Materials

The following are available online at https://www.mdpi.com/2072-6643/12/5/1540/s1. Table S1: Time-point of measures of oxygen uptake (VO_2_), carbon dioxide production (VCO2), respiratory quotient (RQ) and substrate oxidation in the 10 min of the calorimetry test for all groups/supplementation.
