# Supplementary material for: Taurine Supplementation Increases Post-Exercise Lipid Oxidation at Moderate Intensity in Fasted Healthy Males
Source: Nutrients. 2020 May 25;12(5):1540. doi: 10.3390/nu12051540 (PMC7285212; doi:10.3390/nu12051540)
Supplement: Supplementary file 1 [file nutrients-12-01540-s001.pdf]

Supplementary Table S1. Time-point of measures of oxygen uptake (VO_2), carbon dioxide production (VCO_2), respiratory quotient (RQ) and substrate oxidation in the 10 minutes of the calorimetry test for all groups/supplementation.

**VO_2**	**1min**	**2min**	**3min**	**4min**	**5min**	**6min**	**7min**	**8min**	**9min**	**10min**
PLACEBO	0.91±0.2	0.55±0.08 [a]	0.46±0.04 [a,b]	0.44±0.05 [a,b]	0.43±0.06 [a,b,c]	0.4±0.04 [a,b,c,d,e]	0.4±0.05 [a,b,c,d,e]	0.39±0.07 [a,b,c,d,e]	0.37±0.05 [a,b,c,d,e,f]	0.37±0.05 [a,b,c,d,e,f]
TAU 3g	0.93±0.2	0.58±0.09[a]	0.48±0.05 [a,b]	0.45±0.02 [a,b,c]	0.43±0.04 [a,b,c]	0.4±0.04 [a,b,c,d,e]	0.4±0.04 [a,b,c,d,e]	0.39±0.04 [a,b,c,d,e]	0.38±0.04 [a,b,c,d,e]	0.38±0.03 [a,b,c,d,e,f,g]
TAU 6g	1.0±0.1	0.60±0.09 [a]	0.50±0.06 [a,b]	0.47±0.08 [a,b]	0.44±0.05 [a,b,c,d]	0.4±0.05 [a,b,c,d]	0.41±0.05 [a,b,c,d,e,f]	0.40±0.05 [a,b,c,d,e,f]	0.40±0.05 [a,b,c,d,e,f]	0.39±0.05 [a,b,c,d,e,f,g]

**VCO_2**	**1min**	**2min**	**3min**	**4min**	**5min**	**6min**	**7min**	**8min**	**9min**	**10min**
PLACEBO	0.79±0.1	0.49±0.07 [a]	0.41±0.04 [a,b]	0.39±0.05 [a,b]	0.38±0.07 [a,b]	0.36±0.05 [a,b,c,d,e]	0.34±0.03 [a,b,c,d,e]	0.33±0.07 [a,b,c,d,e,f]	0.31±0.05 [a,b,c,d,e,f,g]	0.30±0.05 [a,b,c,d,e,f,g,h]
TAU 3g	0.82±0.2	0.53±0.1 [a]	0.40±0.03 [a,b]	0.40±0.37 [a,b]	0.36±0.03 [a,b,c]	0.34±0.04 [a,b,c,d]	0.33±0.03 [a,b,c,d]	0.32±0.03 [a,b,c,d,e]	0.32±0.05 [a,b,c,d]	0.30±0.03 [a,b,c,d,e,g]
TAU 6g	0.87±0.1	0.52±0.08 [a]	0.42±0.06 [a,b]	0.40±0.07 [a,b,c]	0.36±0.04 [a,b,c,d]	0.36±0.03 [a,b,c,d]	0.34±0.03 [a,b,c,d,f]	0.34±0.04 [a,b,c,d]	0.32±0.04 [a,b,c,d,e,f,g]	0.31±0.03 [a,b,c,d,e,f,g,h]

**RQ**	**1min**	**2min**	**3min**	**4min**	**5min**	**6min**	**7min**	**8min**	**9min**	**10min**
PLACEBO	0.86±0.07	0.90±0.07 [a]	0.88±0.06	0.87±0.06	0.88±0.07	0.90±0.11	0.89±0.13	0.86±0.10 [b,f]	0.83±0.05 [a,b,c,d,e,f]	0.81±0.07 [a,b,c,d,e,f,h,i]
TAU 3g	0.83±0.03	0.88±0.05 [a]	0.88±0.05 [a]	0.90±0.1 [a]	0.86±0.06 [a,d]	0.84±0.06 [b,d]	0.83±0.05 [a,b,c,d,e,f,h,]	0.82±0.04 [b,c,d,e]	0.82±0.06 [b,c,d,e]	0.82±0.05 [b,c,d,e]
TAU 6g	0.82±0.04	0.86±0.05 [a]	0.85±0.06 [a]	0.85±0.06	0.83±0.04	0.83±0.04	0.83±0.04	0.83±0.04 [i]	0.80±0.41 [b,c,d,g]	0.78±0.03 [a,b,c,d,e,g,i]

**Oxidation Lipid**	**1min**	**2min**	**3min**	**4min**	**5min**	**6min**	**7min**	**8min**	**9min**	**10min**
PLACEBO	0.21±0.14	0.09±0.08 [a]	0.09 ±0.05 [a]	0.09±0.05 [a]	0.08±0.06 [a]	0.06±0.08 [a]	0.08±0.08 [a]	0.09±0.06 [a,f]	0.10±0.03 [a,f]	0.12±0.04 [a,c,e,f,h,i]

TAU 3g	0.27±0.09	0.11±0.05 [a]	0.09±0.05 [a]	0.07±0.08 [a,b]	0.10±0.05 [a,d]	0.10±0.04 [a,d]	0.11±0.03 [a,d]	0.12±0.04 [a,d]	0.11±0.04 [a,d]	0.11±0.03 [a,d]
TAU 6g	0.31±0.08	0.14±0.07 [a]	0.12±0.06 [a,b]	0.12±0.07 [a]	0.12±0.05 [a]	0.12±0.04 [a]	0.12±0.04 [a]	0.11±0.04 [a]	0.13±0.04 [a]	0.14±0.04 [a,h]

**Oxidation CHO**	**1min**	**2min**	**3min**	**4min**	**5min**	**6min**	**7min**	**8min**	**9min**	**10min**
PLACEBO	0.66±0.28	0.49±0.18 [a]	0.39±0.13 [a,b]	0.36±0.13 [a,b]	0.37±0.18 [a,b]	0.36±0.21 [a,b]	0.32±0.17 [a,b]	0.29±0.17 [a,b,c,f]	0.23±0.09 [a,b,c,d,e,f,h]	0.19±0.13 [a,b,c,d,e,f,h,i]
TAU 3g	0.57±0.18	0.51±0.18	0.41±0.16 [a,b]	0.43±0.21 [a]	0.32±0.13 [a,b,c,d]	0.27±0.12 [a,b,c,d]	0.24±0.09 [a,b,c,d,e,f]	0.22±0.09 [a,b,c,d,e,g]	0.22±0.14 [a,b,c,d,e,g]	0.21±0.10 [a,b,c,d,e,g]
TAU 6g	0.60±0.25	0.43±0.16 [a]	0.35±0.14 [a,b]	0.32±0.17 [a,b]	0.26±0.08 [a,b,c]	0.26±0.09 [a,b,c]	0.24±0.07 [a,b,c]	0.25±0.06 [a,b,c]	0.19±0.08 [a,b,c,d,h]	0.15±0.06 [a,b,c,d,e,f,h,i]

Min: minute. TAU 3g: 3 grams of taurine. TAU 6g: 6 grams of taurine. RQ: respiratory quotient. Oxidation CHO: carbohydrate oxidation.
Letters represents statistical difference between time-point.
a= vs 1min; b= vs 2min; c= vs 3min; d= vs 4min; e= vs 5min; f= vs 6min; g= vs7min; h= vs 8min, i= vs 9min; j= vs 10min.
vs= versus ($p<0.05$), by ANOVA two way repeated measures. Data expressed as mean and standard deviation (M±SD).
e.g. If letter “a” is in the time 2minutes and 3minutes, this represents that 1minute is statistically different from 2 and 3 minutes.
This representation is only to show differences between time-point in the same group/supplementation.
